# Postoperative outcomes of gastric carcinoma with lymphoid stroma

**DOI:** 10.1186/s12957-020-01878-9

**Published:** 2020-05-21

**Authors:** Kenichi Iwasaki, Takeshi Suda, Yuki Takano, Yuki Ohno, Erika Yamada, Naoto Okazaki, Kosuke Takahashi, Takafumi Watanabe, Yosuke Makuuchi, Yoshihiro Ota, Yoshiaki Osaka, Akiyoshi Seshimo, Kenji Katsumata, Akihiko Tsuchida

**Affiliations:** Department of Gastrointestinal and Pediatric Surgery, Tokyo Medical University, 6-7-1 Nishishinjuku, Shinjuku-ku, Tokyo, 160-0023 Japan

**Keywords:** Gastric carcinoma with lymphoid stroma, Gastric cancer, Lymph node

## Abstract

**Background:**

Gastric carcinoma with lymphoid stroma (GCLS) is a rare subtype of gastric cancer. There have been several reports demonstrating the favorable prognosis of early GCLS without lymph node metastasis (LNM) compared with gastric adenocarcinomas. However, it remains unknown whether advanced GCLS (AGCLS) with LNM has a similar prognosis and clinicopathological features. This study aimed to assess the clinicopathological features of GCLS of all stages.

**Methods:**

We retrospectively assessed 375 patients who were pathologically diagnosed with gastric cancer and underwent curative surgical resection at Tokyo Medical University, Japan, between September 2013 and October 2019. Of these patients, 357 (95.2%) patients were pathologically diagnosed with gastric adenocarcinomas, and 18 (4.8%) patients were diagnosed with GCLS. The GCLS patients (*n* = 18) were compared with the gastric adenocarcinoma patients (non-GCLS patients, control) (*n* = 357) in terms of their clinicopathological features and clinical outcome.

**Results:**

The GCLS patients showed significantly predominant upper gastric locations (*P* = 0.003), lower number of LNM (*P* = 0.01), and better overall survival rate than the non-GCLS patients (*P* = 0.029). The predominant upper gastric locations (*P* = 0.0002), lower number of LNM (*P* = 0.003), and better overall survival rate (*P* = 0.04) were significantly correlated in the AGCLS with LNM patients compared with the advanced non-GCLS with LNM patients. For survival analyses, surgical procedure, tumor location, and numbers of positive LNM were adjusted by 1:1 propensity score matching. After adjustment, the overall survival rate was significantly higher in the AGCLS group than in the advanced non-GCLS group (*P* = 0.03).

**Conclusion:**

AGCLS has distinct clinicopathological features and clinical behavior that are similar to those of early GCLS. AGCLS with LNM patients showed a significantly lower number of LNM and a better survival rate than advanced non-GCLS with LNM patients. To our knowledge, this study is the first report to describe the clinicopathological features of AGCLS.

## Background

Gastric carcinoma with lymphoid stroma (GCLS), which is also known as gastric lymphoepithelioma-like carcinoma, is a rare histological subtype of gastric cancer, the fourth most frequent cancer in the world [1]. It is also called medullary carcinoma or lymphoepithelioma-like carcinoma [2–4]. GCLS accounts for only about 1–4% of all malignant gastric tumors, and up to 80% of the reported GCLS cases are associated with an Epstein–Barr virus (EBV) infection [5–8].

Histologically, GCLS is characterized by a high density of tightly packed tumor cells with extensive lymphocytic infiltrations into the surrounding stroma and the tumor itself [9, 10]. Despite these known histological features, its diagnostic criteria have not been standardized, and its molecular features remain obscure. There have been several reports demonstrating the favorable prognosis of GCLS compared with other gastric adenocarcinomas, as well as its differentiating clinicopathological features [11–16]. With its low frequency of lymph node metastasis (LNM), an expanded indication of endoscopic resection in early GCLS cases has been suggested [17–20]. However, to our knowledge, current information on GCLS including its clinicopathological features, survival outcomes, and treatment remains inadequate with only a few studies owing to its rarity [13]. Moreover, it remains unclear whether advanced GCLS (AGCLS) with LNM patients have a favorable prognosis. Although GCLS is an infrequently encountered subtype of gastric cancer, it is equally relevant to clarify its outcome and clinicopathological features to obtain a better understanding of this rare but important disease.

This study aimed to assess and compare the clinicopathological features and clinical outcomes between GCLS patients and non-GCLS patients, not only in the early stages but also in all the stages, including AGCLS with LNM patients who underwent curative surgical resection in our hospital.

## Methods

We retrospectively assessed 375 patients who were pathologically diagnosed with gastric cancer and underwent curative surgical resection at Tokyo Medical University Hospital, Japan, between September 2013 and October 2019. Of these patients, 357 (95.2%) patients were pathologically diagnosed with conventional differentiated gastric adenocarcinomas with no GCLS morphology (i.e., non-GCLS patients, control), and 18 (4.8%) patients were pathologically diagnosed with GCLS (i.e., GCLS patients). In all patients, curative gastrectomy with lymphadenectomy was routinely performed.

The Japanese Classification of Gastric Carcinoma 15th edition [21] was used to diagnose and examine the resected specimens. The gastric carcinomas were classified into stages according to the Union for International Cancer Control classification 8th edition [22]. GCLS was determined as a poorly differentiated or undifferentiated tumor with prominent lymphoid infiltration on the basis of the 2010 World Health Organization classification guidelines [23].

**Fig. 1:**
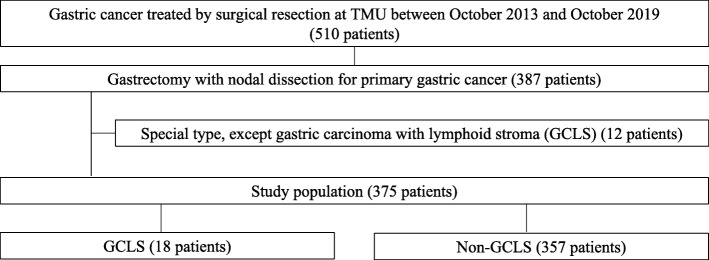
Flow diagram of patients who underwent gastrectomy for gastric cancer between September 2013 and October 2019

The GCLS patients were compared with the non-GCLS patients in terms of clinicopathological features including factors such as age, sex, treatment outcomes, tumor site, macroscopic type, tumor size, tumor depth, LNM, and lymphovascular invasion which were obtained from the medical chart reviews, histology slides, and pathology reports. All patients were followed up for 5 years, or until death if it came earlier. Figure 1 shows the flow diagram of the patients registered in this study.

### Statistical analysis

Quantitative data were expressed as mean ± standard deviation (SD). The Kaplan-Meier method was used to determine the cumulative survival rate. The log-rank test was applied to analyze the correlation between the clinicopathological factors and the survival of GCLS and non-GCLS patients. Propensity score matching was used for 1:1 matching to adjust the difference between the two groups. Selected covariables included age, gender, tumor location, chemotherapy, T stage, LNM, and TNM stage. Statistical analysis was performed using SPSS 13.0. A *P* value of < 0.05 was considered to indicate a statistically significant difference.

## Results

**Fig. 2:**
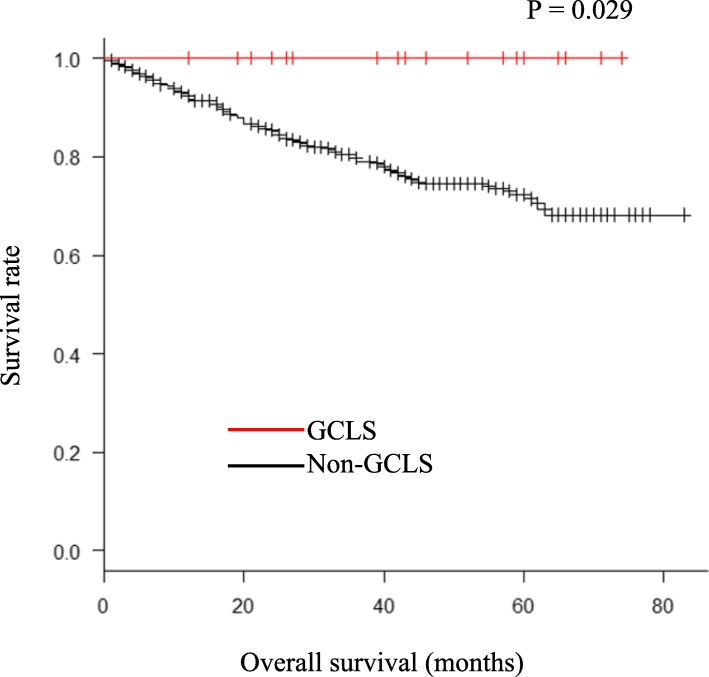
Kaplan–Meier survival curves of GCLS and non-GCLS patients

A comparative summary of the background and clinicopathological features of the GCLS patients and non-GCLS patients is shown in Table 1. The GCLS patients consisted of 14 (77.8%) men and 4 women whose age ranged from 48 to 89 years (mean 71 years). Total gastrectomy was the most common surgical procedure (10/18, 55.6%), followed by distal gastrectomy. As shown in Table 1, there was no significant difference in the tumor diameter between the GCLS patients and the non-GCLS patients (*P* = 0.21), although GCLS was found more in proximal locations (upper third 61.1%/20.2%, *P* = 0.003). Only one patient out of the 18 GCLS patients showed tumor multiplicity (5.6%). The most common macroscopic type was superficial (9/18, 50%). Tumor invasion was most frequent for T1 (mucosa or submucosa). There was no significant difference in the number of dissected lymph nodes per surgical specimen between the GCLS patients and the non-GCLS patients (*P* = 0.53). However, the GCLS patients were associated with a significantly lower number of LNM (*P* = 0.01). There was no significant difference in the lymphatic or vascular invasion and pathological stage between the two groups. Only one patient was EBV-negative in the GCLS group (EBV positivity was not determined in the control group). There was a significant difference in the overall survival rate between the GCLS patients and the non-GCLS patients (Fig. 2, *P* = 0.029). The detailed clinicopathological features of each GCLS patient are summarized in Table 2.

**Table 1:** Background and clinicopathological features of GCLS and non-GCLS patients

		GCLS patients	Non-GCLS patients (control)	*P* value
		*n* = 18 (%)	*n* = 357 (%)	
Age (years, mean ± SD)		71 ± 12.3	72 ± 11.6	0.60
Gender				0.70
	Male	14 (77.8)	263 (73.7)	
	Female	4 (22.2)	94 (16.3)	
Surgical procedures				0.11
	TG	10 (55.6)	108 (30.3)	
	DG	7 (38.9)	221 (61.9)	
	PG	1 (5.6)	19 (7.8)	
Tumor diameter (mm, mean ± SD)		25 ± 26.9	47.2 ± 30.8	0.21
Site of tumor				0.003
	Upper third	11 (61.1)	72 (20.2)	
	Middle third	4 (22.2)	175 (49.0)	
	Lower third	3 (16.7)	110 (30.8)	
Number of tumors				0.92
	Single	17 (94.4)	329 (92.2)	
	Multiple	1 (5.6)	28 (7.8)	
Macroscopic type				0.44
	Superficial	9 (50.0)	190 (53.2)	
	Borrmann I1 (5.6)	19 (5.3)		
	Borrmann II	4 (22.2)	52 (14.6)	
	Borrmann III	4 (22.2)	68 (19.1)	
	Borrmann IV	0 (0)	13 (3.6)	
	Borrmann V	0 (0)	15 (4.2)	
Tumor invasion				0.48
	T0	0 (0)	0 (0)	
	T1	11 (61.1)	184 (51.5)	
	T2	3 (16.7)	37 (10.4)	
	T3	0 (0)	70 (19.6)	
	T4	4 (22.2)	66 (18.5)	
Number of harvested LN (mean ± SD)		52 ± 29.4	47.3 ± 20.5	0.53
LN metastasis				0.94
	Absent	11 (61.1)	218 (61.1)	
	Present	7 (38.9)	139 (38.9)	
Number of positive LN (mean ± SD)		1.39 ± 2.43	3.24 ± 7.8	0.01
Lymphatic invasion				0.33
	ly0	10 (55.6)	154 (43.1)	
	ly1	2 (11.1)	114 (31.9)	
	ly2	4 (22.2)	52 (14.6)	
	ly3	2 (11.1)	37 (10.4)	
Vascular invasion				0.68
	v0	10 (55.6)	180 (50.4)	
	v1	5 (27.8)	100 (28.0)	
	v2	3 (16.7)	55 (15.4)	
	v3	0 (0)	22 (6.2)	
Pathological stage	I	10 (55.5)	196 (54.9)	0.57
	II	5 (27.8)	69 (19.3)	
	III	3 (17.7)	89 (24.9)	
	IV	0 (0)	3 (0.8)	
EBV	Positive	17 (94.4)	-	
	Negative	1 (5.6)	-	

*GCLS* gastric carcinoma with lymphoid stroma, *SD* standard deviation, *TG* total gastrectomy, *DG* distal gastrectomy, *PG* proximal gastrectomy, *LN* lymph node, *EBV* Epstein-Barr virus

**Table 2:** Clinicopathological features of 18 GCLS patients

Case	Age (yrs)	Sex	Location	Macroscopic type	No. of tumors	Tumor size (mm)	pT stage	pN stage	No. of LN+/LN total	ly	v	pStage	Surgical procedure
1	48	M	U	0-IIc	1	14	1	0	0/45	0	0	I	DG
2	64	M	M	1	1	25	1	0	0/67	0	0	I	DG
3	75	M	L	0-IIc	1	13	1	0	0/54	0	0	I	DG
4	55	M	U	0-IIc	3	24	1	2	4/62	0	0	II	TG
5	42	M	U	2	1	25	1	1	2/31	0	1	II	TG
6	63	M	U	3	1	100	4	1	2/83	2	1	III	TG
7	64	M	U	0-IIc	1	44	1	0	0/56	0	0	I	TG
8	74	F	U	3	1	52	4	1	1/48	1	2	III	TG
9	89	M	M	0-IIc	1	16	1	0	0/25	0	0	I	DG
10	72	M	U	0-IIa	1	20	2	1	2/9	2	2	II	TG
11	76	F	L	2	1	50	1	0	0/52	2	0	I	DG
12	84	M	M	2	1	76	2	2	5/24	3	2	II	DG
13	83	M	U	2	1	20	2	0	0/11	2	1	I	TG
14	80	M	U	0-IIc	1	10	1	0	0/34	0	0	I	PG
15	65	F	M	0-IIa	1	13	1	0	0/52	0	0	I	DG
16	65	M	U	3	1	75	4	3a	9/72	3	1	III	TG
17	70	F	U	3	1	55	4	0	0/133	1	1	II	TG
18	75	M	L	0-IIc	1	65	1	0	0/75	0	0	I	TG

*M* male, *F* female, *U* upper third, *M* middle third, *L* lower third, *No.* number, *p* pathological, *LN* lymph node, *ly* lymphatic invasion, *v* venous invasion, *DG* distal gastrectomy, *TG* total gastrectomy, *PG* proximal gastrectomy

A comparative summary of the background and clinicopathological features of AGCLS with LNM patients and advanced non-GCLS with LNM patients (conventional adenocarcinoma with LNM) is shown in Table 3. The AGCLS with LNM patients consisted of 6 (85.7%) men and 1 woman whose age ranged from 42 to 84 years (mean 72 years). Total gastrectomy was the most frequent surgical procedure in the AGCLC with LNM patients (6 of 7, 85.7%), which showed a significant difference compared with the advanced non-GCLS with LNM patients (*P* = 0.03). The tumor diameter of the AGCLS with LNM patients was 61.5 ± 31.5 mm (mean ± SD) whereas that of the advanced non-GCLS with LNM patients was 53.1 ± 31.4 (*P* = 0.51). As also shown in GCLS, the AGCLS with LNM patients showed predominant upper gastric location compared with the advanced non-GCLS with LNM patients (*P* = 0.0002). Of the 7 AGCLS with LNM patients, one patient showed tumor multiplicity (14.3%). The most common macroscopic type and tumor invasion were Borrmann III (3/7, 42.8%) and T4 (3/7, 42.8%), respectively. There was no significant difference in the number of harvested lymph nodes per surgical specimen between the AGCLS with LNM patients and the advanced non-GCLS with LNM patients (*P* = 0.63). However, the AGCLS with LNM patients were associated with a significantly lower number of LNM (*P* = 0.003). There was no significant difference in the lymphatic or vascular invasion and pathological stage between the two groups. All the AGCLS with LNM patients were EBV-positive (EBV positivity was not determined in the advanced non-GCLS with LNM patients).

**Table 3:** Background and clinicopathological features of AGCLS and non-GCLS with LNM patients

		AGCLS with LNM patients *n* = 7 (%)	Advanced non-GCLS with LNM patients *n* = 139 (%)	*P* value
Age (years, mean ± SD)		72 ± 10.7	65 ± 13.7	0.19
Gender				0.57
	Male	6 (85.7)	107 (77)	
	Female	1 (14.3)	32 (23)	
Surgical procedures				0.03
	TG	6 (85.7)	63 (45.3)	
	DG	1 (14.3)	70 (50.3)	
	PG	0 (0)	6 (4.4)	
Tumor diameter (mm, mean ± SD)		61.5 ± 31.5	53.1 ± 31.4	0.51
Site of tumor				0.0002
	Upper third	6 (85.7)	36 (25.9)	
	Middle third	1 (14.3)	57 (41.0)	
	Lower third	0 (0)	46 (33.1)	
Number of tumors				0.92
	Single	6 (85.7)	130 (93.5)	
	Multiple	1 (14.3)	9 (6.5)	
Macroscopic type				0.19
	Superficial	2 (28.6)	33 (23.7)	
	Borrmann I	0 (0)	5 (3.6)	
	Borrmann II	2 (28.6)	34 (24.4)	
	Borrmann III	3 (42.8)	47 (33.8)	
	Borrmann IV	0 (0)	11 (7.9)	
	Borrmann V	0 (0)	9 (6.6)	
Tumor invasion				0.57
	T0	0 (0)	0 (0)	
	T1	2 (28.6)	25 (18.0)	
	T2	2 (28.6)	16 (11.5)	
	T3	0 (0)	47 (33.8)	
	T4	3 (42.8)	51 (36.7)	
Number of harvested LN (mean ± SD)		47.0 ± 27.0	52.2 ± 21.4	0.63
Number of positive LN (mean ± SD)		3.57 ± 2.76	8.28 ± 10.7	0.003
Lymphatic invasion				0.76
	ly0	2 (28.6)	8 (5.8)	
	ly1	1 (14.2)	59 (42.4)	
	ly2	2 (28.6)	37 (26.6)	
	ly3	2 (28.6)	35 (25.2)	
Vascular invasion				0.8
	v0	1 (14.2)	26 (18.7)	
	v1	3 (42.9)	54 (38.9)	
	v2	3 (42.9)	42 (30.2)	
	v3	0 (0)	17 (12.2)	
Pathological stage				0.41
	I	1 (14.2)	17 (12.2)	
	II	3 (42.9)	33 (23.8)	
	III	3 (42.9)	87 (62.6)	
	IV	0 (0)	2 (1.4)	
Survival after surgery		44 ± 16.3	28.2 ± 21.3	0.04
(Months; mean ± SD)				
EBV	Positive		7 (100)	-
	Negative	0 (0)	-	

*GCLS* gastric carcinoma with lymphoid stroma, *SD* standard deviation, *TG* total gastrectomy, *DG* distal gastrectomy, *PG* proximal gastrectomy, *LN* lymph node, *EBV* Epstein-Barr virus

**Fig. 3:**
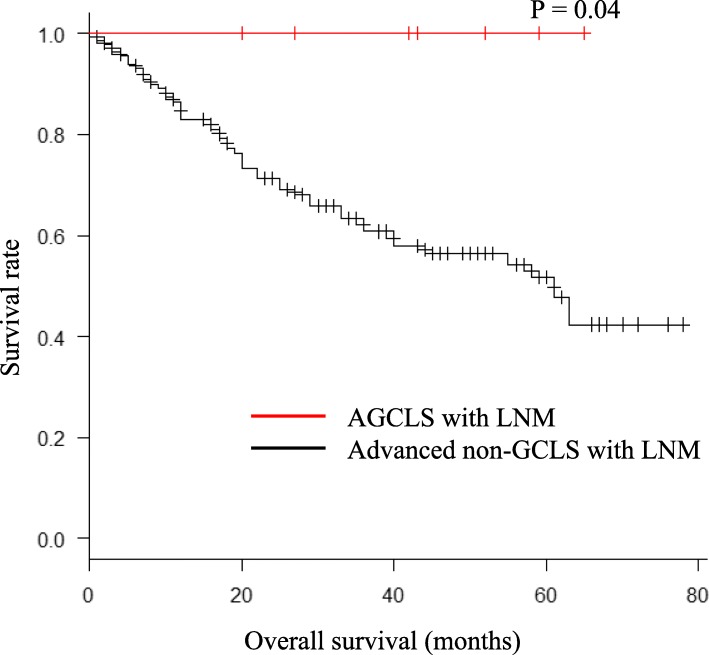
Kaplan–Meier survival curves of AGCLS with LNM patients and advanced non-GCLS with LNM patients

**Fig. 4:**
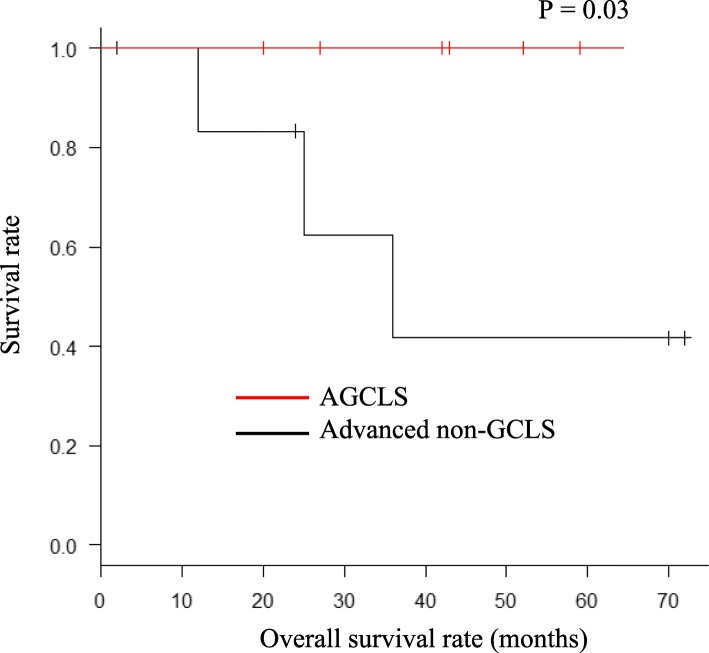
Overall survival rates after adjustment of location, surgical procedure, and positive LNM using propensity score matching for 1:1 matching

There was a significant difference in the overall survival rate between the AGCLS with LNM patients and the advanced non-GCLS with LNM patients (Fig. 3, *P* = 0.04). For the survival analyses, surgical procedure, location, and positive LNM were adjusted by 1:1 propensity score matching. After adjustment, the overall survival rate was significantly higher in the AGCLS group than in the advanced non-GCLS group (Fig. 4, *P* = 0.03).

## Discussion

GCLS, also called gastric lymphoepithelioma-like carcinoma, is a rare subtype of gastric carcinoma that shows distinct clinical characteristics compared with conventional adenocarcinoma. In the present study, 18 patients (4.8%) of the 375 surgically treated gastric carcinoma patients were identified as having GCLS. This frequency is nearly similar to those of previous reports corresponding to 1–4% of all gastric carcinomas [6, 24, 25]. Although the underlying reason remains unclear, earlier studies showed that GCLS has a favorable prognosis with a low LNM rate [6, 10, 20, 26, 27].

Consistent with previous reports [16, 28, 29], in our series, we found that the GCLS patients showed predominant upper gastric locations and were associated with a significantly lower number of LNM than the non-GCLS patients (conventional adenocarcinoma). Moreover, there was a significant difference in the overall survival rate between the GCLS patients and the non-GCLS patients. To date, several studies have reported that early GCLS has unique features with an extremely low frequency of LNM [20, 30]. It has also been reported that the increase in the number of tumor-infiltrating lymphocytes, which reflects the host immune response to tumor cells, was associated with improved survival [31, 32]. The LNM rate of GCLS patients in the present study was 38.9% (7/18 patients).

Shin et al. reported the clinicopathological features of 70 early GCLS patients and showed an extremely low rate of LNM, as well as the tendency for the macroscopic type, tumor location, and tumor size [30]. With its low likelihood of LNM, endoscopic resection has been suggested as an alternative treatment option for early GCLS patients [17–19]. However, despite the favorable prognosis of early GCLS, it remains unclear whether AGCLS also shows a favorable prognosis and similar clinicopathological features. In the present study, we found that among the AGCLS with LNM patients, total gastrectomy was the most frequent surgical procedure (6/17, 85.7%), which showed a significant difference compared with the advanced non-GCLS with LNM patients (*P* = 0.03). Similarly to the GCLS patients, the AGCLS with LNM patients showed predominant upper gastric locations compared with the advanced non-GCLS with LNM patients (*P* = 0.0002). Another finding was that, among all the patients with LNM, the AGCLS patients were associated with a significantly lower number of LNM even though there was no difference in the number of LNs harvested and examined (*P* = 0.003). There was no significant difference in the lymphatic or vascular invasion and pathological stage between the two groups. All patients in the AGCLS group were EBV-positive (EBV positivity was not determined in patients in the control group). As shown in the Kaplan-Meier survival curve in Fig. 3, the overall survival rate of the AGCLS with LNM patients was significantly higher than that of the advanced non-GCLS with LNM patients (*P* = 0.04). As this is apparently the first report to show the long-term follow-up of AGCLS with LNM patients, this finding provides a better understanding of this rare disease. As the surgical procedure, location, and number of LNM were significantly different, we adjusted each factor by 1:1 propensity score matching to assess the prognosis and survival of AGCLS with LNM patients and to compare them with those of advanced non-GCLS with LNM patients.

Interestingly, we found that even after adjustment, the overall survival rate was significantly higher in the AGCLS group than in the advanced non-GCLS group (Fig. 4, *P* = 0.03). Some studies have reported that less LNM is a factor for good prognosis in GCLS [7, 15, 29]. However, the present findings indicate that further studies are warranted to fully clarify the clinical and histological features of GCLS that are associated with a good prognosis [4, 18, 22].

Kim et al. reported the association between the sizes of the gastric carcinoma and LNM [33]. In their study, the tumor size was the only significant risk factor for LNM in the analysis of 574 patients. However, in the present study, there was no significant difference in the size of the tumor between the AGCLS with LNM patients and the advanced non-GCLS with LNM patients.

In their report of 40 GCLS patients, Lim et al. (2018) suggested that endoscopic resection without LN dissection could be an alternative option for early gastric cancer with lymphoid stroma patients [34]. Our results of 18 GCLS patients also showed an extremely good survival rate after surgery, although one patient had recurrence even if the tumor stage was IB and with only one LNM. According to this finding, surgical resection with radical LN dissection may contribute to the good prognosis of GCLS patients even if they have a low risk of LNM. On the other hand, we cautiously suggest that GCLS may be a good candidate for not performing adjuvant chemotherapy, which is a standard therapy for stages II and III gastric cancer in Japan.

Our study has several limitations as follows. The analysis had a retrospective nonrandomized design, the sample size of the GCLS group was small, and the follow-up period after surgery was relatively short in some cases. There were no patients with stage IV tumor in the GCLS group. This was likely due to the small sample size which could have led to some biases. Evaluation of the EBV status of the non-GCLS patients was not performed. Moreover, it remains unclear whether the good prognosis of the GCLS patients is due to the role of EBV.

## Conclusion

AGCLS with LNM has distinct clinicopathological features and clinical behavior that are similar to those of early GCLS. AGCLS with LNM patients showed a significantly lower number of LNM and a better survival rate than advanced non-GCLS with LNM patients. An accurate diagnosis and recognition of GCLS, as well as detailed studies of GCLS based on a larger number of patients are warranted to a further enhance the efficacy of existing treatments or to developed novel treatments for GCLS.

## Data Availability

The datasets generated and/or analyzed during the current study are available from the corresponding author on reasonable request.

## Abbreviations

GCLS: Gastric carcinoma with lymphoid stroma
LNM: Lymph node metastasis
AGCLS: Advanced GCLS with LNM
EBV: Epstein–Barr virus
